# The role of community volunteers in PMTCT programme: Lessons from selected sites in Zambia to strengthen health education on infant feeding and follow-up of HIV-positive mother-infant pair

**DOI:** 10.4102/phcfm.v10i1.1665

**Published:** 2018-06-18

**Authors:** Alice Ngoma-Hazemba, Busisiwe P. Ncama

**Affiliations:** 1 School of Public Health, University of Zambia, Zambia; 2 School of Nursing and Public Health, Howard College Campus, University of KwaZulu-Natal, South Africa

## Abstract

**Background:**

A global debate surrounding health care delivery at the lowest level of the community has aroused interest among researchers. In settings where skilled health workforce is scarce, the community relies on volunteers to provide care.

**Aim:**

To explore the role of community-based volunteers (CBVs) and their perspectives on human immunodeficiency virus (HIV) and infant feeding to gain insights into the implementation of prevention of mother-to-child transmission (PMTCT) interventions at community level.

**Setting:**

The study was conducted in Lusaka using Ngombe and Chelstone health facilities to recruit participants. Fieldwork took place from January 2014 to September 2014.

**Methods:**

An exploratory descriptive qualitative study employing focus group discussions was conducted with CBVs. Convenient sampling was used to recruit 10 participants from each site. All transcribed interviews were imported into the Nvivo 10 for open coding and analysis.

**Results:**

Although the role of community volunteers was to support and teach mothers on infant feeding in relation to HIV, the known cultural norms and practices had a bearing on how they tailored their information on breastfeeding to mothers. However, their link of the community to the health facilities cannot be overemphasised in these settings.

**Conclusion:**

The role of community volunteers in PMTCT interventions can be strengthened by improving their training through use of appropriate educational materials and support of required resources. Lessons from these sites can inform future research to design community-based interventions and develop health education materials that are sensitive to cultural norms and practices in this and similar settings.

## Introduction

Although there have been improvements over the past decade, the prevalence of human immunodeficiency virus (HIV) among adults aged 15–59 years in Zambia is 12.3%; 14.9% among women and 9.5% among men, and this corresponds to approximately 980 000 people living with HIV (PLHIV) within the same age group.^1,2^ In settings with the highest burden of HIV among women in the reproductive age and with a high number of new paediatric infections, the uptake of health facility based care by pregnant women remains low.^3^ Hence, poor retention in the prevention of mother-to-child transmission (PMTCT) programme continues to limit the impact of care for mothers living with HIV and their exposed infants.

HIV-positive mothers practice infant feeding in communities where they live and work. Therefore, in these settings, the continuum of care for mother-infant pair may require a package of interventions that are sensitive to the needs of the community at the lowest level.^4^ In Zambia, 60% of the population live in the rural areas, where access to health services is a challenge because of geographical barriers such as rivers, inaccessible roads and wildlife reserves, just to mention a few,^2,5^ and in most health facilities, staffing levels are still very low or not available.^6^

The ability of HIV-positive mothers to successfully achieve a desired feeding method is significantly influenced by the support provided through formal health services, social support systems and other community-based groups. Respecting the mothers’ independence would be to empower them with sufficient information about different options of infant feeding so that they can make an independent informed decision and will know where to seek help and further information, if required.^7,8,9^ Therefore, some community-based interventions have been identified as essential components in improving knowledge and uptake of PMTCT services especially in low-resource settings^3,10^ and the World Health Organization (WHO) recommends building the capacity of lay counsellors and support groups in promoting breastfeeding to enable HIV-positive mothers to practice safer feeding for their exposed infants.^11^ Hence, the environment within which mothers practice infant feeding has a role to play in child health outcomes.

Some countries in sub-Saharan Africa have embraced community structures to enhance health service delivery to the lowest level and provide a basic package of care by community volunteers^12,13^; however, more is required to enhance the available opportunities. In Zambia, currently, the Ministry of Health (MoH) has embraced Safe Motherhood Action Groups (SMAGs), a description used for community care givers such as community health workers (CHWs), traditional birth attendants (TBAs), lay counsellors, treatment supporters, community-based commodity distributers and others. Unlike more specialised health care providers, these people are often readily available where the need is greatest and can be efficiently and effectively trained and recruited to provide health education and support mothers in PMTCT programmes within their local communities. More importantly, these community groups can effectively engage mother-infant pairs and partners both at health facilities and within the community.^3,14^

Although, health care systems may engage community volunteers to provide basic health services to the lowest level of communities, research in such approaches is required for broader understanding of their effectiveness in resource-limited settings.^10,15,16^ The question we asked for this study was the following: how does the training on lay counselling and infant feeding empower community volunteers to provide health education information on breastfeeding and support HIV-positive mother-infant pair in PMTCT programmes?

## Research methods and design

### Overall aim of the study

To explore the role of community-based volunteers (CBVs) and their perspectives on HIV and infant feeding and gain insights into the implementation of PMTCT interventions at community level.

### Study design

This exploratory descriptive qualitative study was conducted as part of the main research titled: *HIV and infant feeding: choices and decision-outcomes in the context of PMTCT among HIV-positive mothers in selected sites in Zambia*.^17^ For the main project, data collection was done through individual interviews with mothers, health care workers and focus group discussions (FGDs) with HIV-positive men and CBVs. Therefore, this article addresses the perspectives of community volunteers regarding their role as lay counsellors in conducting health education on infant feeding and follow-up of mother-infant pairs. For CBVs, the FGDs as a qualitative approach was appropriate because breastfeeding is universally regarded as a way of fulfilling motherhood and hence using conversations was intended to allow a debate at the community level on how they contextualised infant feeding in relation to HIV.

### Study setting

This study was conducted in an urban setting of Lusaka district and two health facilities (Chelstone and Ngombe) were used to recruit study participants. These are sites within the Lusaka district which provide 24 h maternal, newborn and child health (MNCH) services and outpatient care. These are also sites for the Ministry of Health programmes on PMTCT of HIV and antiretroviral therapy (ART).

### Study participants

The study participants were mainly CBVs (*n* = 20). As CBVs, they were trained as lay counsellors to conduct health education on infant feeding in the context of PMTCT and support HIV-positive mothers in their communities. For each site, all the 10 CBVs participated in the FGDs. They were invited because they were the only ones assigned to the health facilities at the time of conducting this study.

### Sampling

Convenient sampling methods were used to recruit all the 20 participants. For each health facility, there were 10 CBVs (Table 1) attached to the MNCH departments.

**TABLE 1:** Participant information.

ID no.	Sex	Age	Marital status	Training and/or skills	Education
CH1	F	35	Married	Lay counselling and/or BF&PMTCT	Grade 12
CH2	F	45	Married	Lay counselling and/or BF&PMTCT	Tertiary
CH3	F	30	Single	Lay counselling and/or BF&PMTCT	Grade 11
CH4	F	52	Widow	Lay counselling and/or BF&PMTCT	Grade 10
CH5	M	58	Married	Lay counselling and/or BF&PMTCT	Grade 8
CH6	M	45	Married	Lay counselling and/or BF&PMTCT	Grade 12
CH7	F	49	Single	Lay counselling and/or BF&PMTCT	Grade 7
CH8	F	37	Married	Lay counselling and/or BF&PMTCT	Grade 8
CH9	M	40	Married	Lay counselling and/or BF&PMTCT	Tertiary
CH10	F	35	Married	Lay counselling and/or BF&PMTCT	Grade 6
NG1	F	33	Married	Lay counselling and/or BF&PMTCT	Grade 9
NG2	F	47	Married	Lay counselling and/or BF&PMTCT	Grade 10
NG3	F	49	Married	Lay counselling and/or BF&PMTCT	Grade 7
NG4	F	47	Married	Lay counselling and/or BF&PMTCT	Grade 9
NG5	M	39	Single	Lay counselling and/or BF&PMTCT	Grade 11
NG6	F	40	Widow	Lay counselling and/or BF&PMTCT	Grade 9
NG7	M	43	Married	Lay counselling and/or BF&PMTCT	Grade 12
NG8	F	48	Married	Lay counselling and/or BF&PMTCT	Grade 10
NG9	F	44	Married	Lay counselling and/or BF&PMTCT	Grade 7
NG10	M	69	Married	Lay counselling and/or BF&PMTCT	Grade 9

CH, Chelstone health facility; NG, Ngombe health facility; BF, breastfeeding; PMTCT, prevention of mother-to-child transmission; F, female; M, male.

### Interview guide

A semi-structured focus group guide was used to conduct the discussions. The questions focused on the knowledge of mother-to-child transmission (MTCT) of HIV, the role of the community volunteers in PMTCT, known cultural norms and practices of breastfeeding, and challenges and recommendations for future PMTCT programmes.

### Data collection

The fieldwork was conducted from January 2014 to September 2014 by the principal investigator (PI), the first author, and two research assistants (RAs). The RAs who were nurse midwives with additional training in HIV counselling and PMTCT management conducted all the interviews under the supervision of the PI. The interviews were conducted in the primary languages spoken by the participants (Cibemba and Cinyanja). Data saturation was reached when no more new information was elicited from the participants.

The FGDs were held at the health facilities because all the participants felt it was convenient for them as they were coming from different locations of the communities. However, privacy was ensured by holding the FGDs in the rooms situated away from health facility activities. During the discussions, refreshments were provided and at the end of the session and all the participants were reimbursed with transport money to go back to their homes.

### Data management

Two other RAs were recruited to transcribe the interviews verbatim from the primary languages spoken by the participants into English. Training of all RAs was conducted by the PI and Nvivo Software specialist in one week regarding the transcription and data coding. All the transcripts were imported into Nvivo 10 for coding and analysis based on the emerging themes. The open-coding process helped identify key themes that formed the initial coding structure, which was evolving as analysis progressed. After reviewing more data through open coding, recoding and categorising, the final coding structure was developed. The integrated data on each theme were analysed for varying and similar perspectives on each theme. For this study, four major themes emerged that gave meaning for the understanding of the role of community volunteers in PMTCT programmes.

### Data analysis

The analysis was based on participant construction of knowledge regarding PMTCT in the context of HIV. The constructivist perspective states that knowledge is socially and culturally constructed and proposes that reality cannot be discovered because it does not exist until it is created by individuals. It further states that individuals create their own realities or truths based on a given experience. Constructivism is a theory of knowledge construction and the enquiry aims of this paradigm are oriented to the production of reconstructed understandings.^18^ Therefore, knowledge in this context is considered a social product. The participants in this study constructed their own knowledge based on the understanding of information they received during training as CBVs. Thus, we built a consensus of this understanding both from participants’ perspectives and our experience with PMTCT programmes. Thus, the integrated data on each theme was analysed for varying and similar perspectives to draw conclusions. This not only produced comprehensive findings on each theme but also provided a complex analysis of perspectives. Furthermore, refining of subthemes for logical flow of information led to construction of the total picture of community volunteering. In all the themes, participants’ own words (quotes) were linked to the categories in the subthemes.

## Ethical consideration

This sub-study was part of a bigger research titled: *HIV and infant feeding: choices and decision-outcomes in the context of prevention of mother-to-child transmission among HIV-positive mothers selected sites in Zambia*.^17^ The study was grunted ethical approval by the Humanities and Social Sciences Research Ethics Committee of the University of KwaZulu-Natal in South Africa (HSS/0104/013D) and the Biomedical Research Ethics Committee of the University of Zambia (Reference No. 016-11-13). Voluntary participation was accorded with written consent. No identifiers were used to ensure confidentiality, and interviews were conducted at a place convenient to the participants. At any given time, participants were free to withdraw from the study and continuity of care for mothers and volunteer work for community volunteers was assured.

## Findings

Four major thematic areas emerged from this study: the understanding of mother-to-child transmission of HIV (MTCT) of HIV, the role of community volunteers in PMTCT, cultural norms and practices at variance with breastfeeding in the context of PMTCT and challenges faced by CBVs in their work.

### The understanding of mother-to-child transmission of human immunodeficiency virus

This subtheme highlights how the CBVs described HIV and infant feeding in the context of PMTCT:

‘What I know is that the baby can get HIV from the mothers’ milk during breastfeeding. It happens if in the first six months the baby is given other foods before the intestines become strong. The baby will have sores and when breastfeeding the virus in the milk can easily infect the baby.’ (Chelstone health facility, CH 5, male, 58 years)

Another participant added:

‘If a mother has sores on her breasts and the baby is breastfeeding, the baby can get infected with HIV.’ (Chelstone health facility, CH 8, female, 37 years)

### The role of community volunteers in prevention of mother-to-child transmission

The participants described their role in five different ways: support mothers to involve their partners or spouses in PMTCT, teach mothers about infant feeding in the context of PMTCT, teach and support mothers during breastfeeding, conduct outreach programmes and a link of the health facility to the community. These roles are described as subthemes with participant quotes:

#### Support mothers to involve their partners or spouses in prevention of mother-to-child transmission

Men in these settings rarely get involved in Maternal Newborn Health services. The CBVs were trained to encourage and support women to bring along their spouses or partners during antenatal care (ANC) and subsequently be part of the infant feeding. For instance, consistence in taking antiretroviral (ARV) was considered a couple’s responsibility. A participant explained:

‘We assist mothers to involve their husbands during the antenatal care. By so doing, they will know how to prevent the baby from contracting the HIV virus. Husbands can be reminding their wives to take the medicine (ARVs), and will know for how long the mother should breastfeed the baby.’ (Chelstone health facility, CH 3, female, 30 years)

#### Link the community with the health facilities

The CBVs live in the same communities with women and their families; therefore, they link them with health facilities. Some of the responses were as follows:

‘We work with nurses because we refer clients to them from the community. (Chelstone health facility, CH 6, male, 45 years)

‘We go in the community and find clients. Then we come to the nurses and seek advice. They either tell us to bring the clients to the clinic …’ (Chelstone health facility, CH 2, female, 45 years)

#### Conduct home visits and participate in outreach programmes

The participants added to their list of activities home visits to clients who did not show up for reviews and refills of ARVs and participating in outreach programmes:

‘Through outreach programmes we get mothers that are lazy to come to the clinic for under 5 and then we capture them to get results for HIV testing for their babies andwe give immunisations.’ (Chelstone health facility, CH 6, male, 45 years)

‘For follow-up of mothers, we get guidelines from the nurses because they are the ones having the information in their register. They are the ones who tell us who is not coming for review and then we follow them and bring them to the health facility.’ (Chelstone health facility, CH 6, male, 45 years)

‘…after we conduct home visits in the community, we give the nurses reports on types of clients that we find, what information we have given them, the problems they face and those who have promised to come to the health facilities.’ (Chelstone health facility, CH 2, female, 45 years)

#### Teach mothers on infant feeding in the context of prevention of mother-to-child transmission

The finding that CBVs were aware of the importance of informed choices regarding infant feeding in relation to PMTCT, shows that their primary focus was to know guidelines. One participant explained:

‘We teach mothers about two options: exclusive breastfeeding and breastfeeding as well as formula and we let them choose the method they desire …’ (Chelstone health facility, CH 3, female, 30 years)

Other responses relating to infant feeding were the following:

‘Yes, when we have health talks for antenatal mothers, we talk about how a mother should protect herself and the baby. We talk about exclusive breastfeeding from 0 to 6 months, we talk about children who are HIV-positive and negative. So, when a mother delivers, she will know what to choose, and how she should take care of her baby.’ (Ngombe health facility, NG 9, female, 44 years)

‘The choice to exclusively breastfeed is not because of money. We tell mothers the benefits that are found in breast milk compared with formula…Colostrum, the first milk, which is like the first immunisation for the child is not there in the formula …’. (Ngombe health facility, NG 7, male, 43 years)

‘For mothers who choose formula and they work, we also let them know that the bottle is not safe because when they are not at home they don’t know how the bottles are kept and if they are washed properly…then the baby may continue having diarrhoea. If they can manage to express the milk from the breast … feeder cups are easy to keep and wash … breastmilk is cheap and a mother walks with it.’ (Ngombe health facility, NG 3, female, 49 years)

#### Teach mothers about breastfeeding in the context of prevention of mother-to-child transmission

For mothers who choose to breastfeed, the CBVs reported that they provide support during early stages of infant feeding. The CBVs were aware that some mothers did not want to breastfeed because of fear of infecting their babies with HIV:

‘In the past if the mother is HIV-positive, she would refuse to breastfeed the baby for fear that the baby might get HIV and children used to die. So, we help mothers by teaching them to breastfeed until the baby is 24 months. For protection there is medicine (ARVs) that is used to protect the baby. We help them to exclusively breastfeed and when the baby turns 6 months, they introduce other foods to help the baby to grow, prevent diseases and give strength.’ (Ngombe health facility, NG 10, male, 69 years)

Another participant added:

‘We encourage exclusive breastfeeding from 0 to 6 months for a woman who is either HIV-positive or negative to breastfeed without mixing with other foods until the baby turns 6 months.’ (Chelstone health facility, CH 4, female, 52 years)

Furthermore, they reported teaching mothers about the breastfeeding technique:

‘Another thing is we help them on how to hold the breast when breastfeeding the baby because some hold breasts like a scissors and they squeeze the breast for the milk to come out. But we help them on how to hold the breast and when the mother breastfeeds and the baby finishes the milk, then she moves to the other breast. If the mother breastfeeds the baby like that the nipple cannot have sores and the baby can be protected.’ (Ngombe health facility, NG 10, female, 69 years)

A male participant added:

‘When a mother is found HIV-positive during pregnancy and is given medicine (ARVs), we encourage them to breastfeed without mixing with any other foods or liquids because everything is in the milk unless the doctor says you give medicine. While the baby is on treatment with the mother, at six weeks the baby is brought to be tested at the hospital. Then the test is repeated at six months, at one year and again at one year six months. When the baby is not found to have HIV at one year six months, then the child is safe. When they come to wean the child at two years they stop breastfeeding, we encourage them to give the medicine for a whole week before they stop giving medicine.’ (Ngombe health facility, NG 7, male, 43 years)

While their main focus was health education, the CBVs were concerned that in some instances the mothers did not appear to comprehend the health promotion messages; hence, variations were proposed such as the use of drama:

‘The understanding level of people in the community differs. Some understand through word to word but for some it’s through sketches and drama. There is a need that once in a while we go in the community to conduct drama so that people become fully aware of PMTCT.’ (Chelstone health facility, CH 3, female, 30 years)

### Cultural norms and practices at variance with breastfeeding in the context of prevention of mother-to-child transmission

During the discussions, known cultural practices were highlighted. Much as they were trained to conduct various activities at community level, the participants were also part of the community that embraces traditional norms and practices related to breastfeeding. The following subthemes describe what the participants knew and to a certain extent practiced and supported. It is, therefore, assumed that their roles could have been compromised between what they were expected to do and what they believed in themselves.

#### Using indigenous medicine to protect the baby from diarrhoea

Although participants were advised to encourage mothers to breastfeed their babies on demand, they were challenged with the mothers’ beliefs that doing so in the presence of other breastfeeding mothers would expose their babies to contract diarrhoea (chibele). This finding might entail that in desperate situations, mothers could opt to mix breastfeeding with other liquids as well. Some responses were as follows:

‘Another reason why mothers do not want to breastfeed in public is that some women wear around their waists indigenous medicine tied as a string or on the baby’s wrist to protect the baby from diarrhoea (chibele) and the baby may die if the mother has not protected her child.’ (Chelstone health facility, CH 7, female, 49 years)

‘When a mother comes for BCG and the baby starts to cry, she will leave the group to hide and breastfeed. So, mothers do not breastfeed in the presence of other mothers because they fear ‘chibele’ (diarrhoea).’ (Chelstone health facility, CH 3, female, 30 years)

#### The first milk (colostrum) is dirt

Colostrum, the first milk that comes out at initiation of breastfeeding, is regarded to be dirty and not suitable for the baby:

‘The first milk is dirty, yes, and if the mother’s breastmilk stays for a long time it goes bad and the mother may start giving the child gripe water (off the counter medicine) to clean the stomach.’ (Chelstone health facility, CH 5, male, 58 years)

In addition, another participant said that women believe that breastmilk could go sour if the baby does not breastfeed for some time:

‘… like last time my neighbour made the baby stop breastfeeding at nine months because they left the child and stayed away for one week. When they came back home they asked for money to buy sugar to add to porridge for the child. I asked, but why are you not giving the breast? She answered that because I have stayed for one week and the child has stopped breastfeeding… So I talked to the woman and that is how the baby was put back on the breast until it was two years without having diarrhoea or getting sick. So now that same woman even teaches others near where we live.’ (Ngombe health facility, NG 9, female, 44 years)

#### Delay in initiation of breastfeeding

Late initiation of breastfeeding was practiced when a mother lost her previous baby to allow her to wash the breasts with indigenous medicine. It is assumed that the quotes below suggest that the CBVs were in support of the practice:

‘When a woman had a miscarriage for the previous pregnancy, when she delivers she does not breastfeed until she washes the breasts with traditional herbs, otherwise the new-born baby will also die.’ (Chelstone health facility, CH 2, female, 45 years)

This practice has been passed from generation to generation by elders in the family:

‘When their mothers or grandmothers or in-laws tell them that when this child is born, they should not breastfeed at the clinic, she will not breastfeed but will give other liquids to the baby… ’ (Chelstone health facility, CH 1, female, 35 years)

#### Abrupt cessation of breastfeeding

The participants equally supported abrupt cessation of breastfeeding, pointing out that it is practiced when the mother realises that she is pregnant:

‘The other thing is that if a woman is breastfeeding and suddenly finds out that she is pregnant, she will have to remove the baby from the breast immediately. The fear is that the baby in the womb will share the milk with the breastfeeding baby, and the baby will have diarrhoea and vomiting, or even die.’ (Chelstone health facility, CH 4, female, 52 years)

#### Wet nursing

In some communities, the findings showed that wet nursing is still practiced when a mother to a new born baby dies. Some of the expressions were as follows:

‘… when the mother of a one- or two-day old baby dies, a question of custody is raised and they ask for one having no baby. They make tattoos on her so that she can be able to breastfeed the baby. If the baby is like four months, they just give the baby to someone already breastfeeding so that she breastfeeds it together with her child.’ (Chelstone health facility, CH 4, female, 52 years)

### Challenges faced by community-based volunteers in their volunteer work

The following subthemes describe the limitations that the CBVs faced in achieving their roles. Lack of recognition by the health facility staff, lack of money, loss to follow-up and defaulting clients were some of the challenges they faced in their volunteer work.

#### Lack of recognition by the health facility staff

Despite their self-reported role in the PMTCT programme, it was apparent that CBVs felt unrecognised by the health facility staff:

‘As volunteers we have many ideas to contribute to PMTCT programme but what we need is recognition at the health centres.’ (Ngombe health facility, NG 2, female, 47 years)

#### Lack of money

Considering that distances covered to reach the different families in their catchment communities were far apart, the participants highlighted that additional support was needed (in this case, transport and money to use to buy food on such errands):

‘For the programme to reach the community there is a need for transport and allowances for food. This will enable us to sensitise more people, especially difficult men…’ (Chelstone health facility, CH 6, female, 45 years)

Another participant added:

‘For the programme to run smoothly in the community there is a need for transport and lunch allowances. This will encourage more people to participate in voluntary work.’ (Chelstone health facility, CH 6, female, 45 years)

#### Loss to follow-up and defaulting mothers

Working at the community level, the CBVs sometimes encountered difficulties in following up the mothers on their registers because mothers gave wrong addresses:

‘Most of the time we emphasise when the baby is born after six weeks the baby should be tested for HIV and if after one month the results are not collected, we tell the mother to go and get the results. Also, the under-five card shows how the baby is being fed and we tick. Previously we used to do follow-ups on the mothers who did not collect the results because the register showed information for us to check.’ (Chelstone health facility, CH 6, male, 45 years)

However, another participant highlighted that mothers started giving wrong addresses:

‘The reason why we stopped is because people give us wrong addresses and it is very difficult to locate them.’ (Chelstone health facility, CH 3, female, 30 years)

Some mothers defaulted completely:

‘Sometimes we have defaulters who give wrong contact address and when we go there you find that it is another person or they don’t have anyone who delivered. So, we encourage them to give the correct house numbers or the correct phone numbers. Sometimes when you call the number it’s a wrong phone number.’ (Chelstone health facility, CH 7, female, 49 years)

## Discussion

Barriers that hinder uptake of PMTCT interventions occur at community level where HIV-positive mothers live and feed their exposed infants. Therefore, programmes that do not extend beyond health facilities and do not engage the community might fail to achieve coverage for prevention of vertical transmission of HIV in this and similar settings.^3,19^ The known community groups involved in the achievement of PMTCT outcomes are diverse and include, among others, CHWs, peer counsellors, CBVs, TBAs, mentor mothers, and traditional and religious leaders.^14^ Unlike trained health care providers, these volunteers are often readily available at the lowest level of the community and can be efficiently and effectively trained to support strategies in the prevention of vertical transmission of HIV.^20^ The aim of this study was to explore the role of CBVs and their perspectives on HIV and infant feeding to gain insights into the implementation of PMTCT interventions at community level.

The themes presented in this study affirm that when supported with skills and health education materials on promotion of breastfeeding for HIV-positive mothers, CBVs can be a comprehensive package that can be strengthened to reduce the risk of poor feeding practices for exposed infants. However, additional strategies such as mobile phone-based reminders may improve the community follow-up package as observed in similar settings.^21^ Furthermore, in some communities CBVs have limited education to undertake such roles,^13^ but in this study, all of them were able to read and write and could articulate well the subject under discussion. However, their deep-rooted cultural beliefs and practices had a bearing on how they contextualised and communicated information on breastfeeding practices that were at variance with standard infant feeding guidelines. In this and similar cultures in the sub-Saharan Africa, breastfeeding is a cultural norm; however, certain practices surrounding infant feeding pose a risk of MTCT of HIV.^22,23,24^ Some notable and identified risk practices in this study were late initiation of breastfeeding, mixed feeding with water and other fluids and use of indigenous medicines, regarding colostrum as dirt and wet nursing, among others.^8,25,26^ Late initiation of breastfeeding, for instance, may be associated with mixed feeding for HIV-exposed infants and poses a high risk of post-natal vertical transmission of HIV.^27^ In addition, late initiation of breastfeeding may interfere with exclusive breastfeeding practice despite its well-recognised importance.^24,28^ Therefore, health promotion messages aimed at behavioural change regarding breastfeeding should aim at dispelling the beliefs and practices that have a bearing on optimal breastfeeding practices while endeavouring to build on the ones that have a protective effect on infant and child well-being.^29,30^

Given that the CBVs could comprehend information on PMTCT and they lived in communities they served, they were well placed to gain the confidence of the women and promote behavioural change regarding safe breastfeeding practice. Therefore, embracing best cultural practices and modifying those that pose a risk to MTCT of HIV can be negotiated through the CBVs. In this regard, the value of colostrum could be contextualised based on local knowledge, understanding and experiences.

Another practice identified in this study was that of mothers’ persistence not to breastfeed in public places such as the health facilities on suspicion that their fellow breastfeeding mothers were using protective indigenous medicines on their children to prevent diarrhoea (chibele). In public health, this practice has a protective effect against spread of diarrhoeal diseases through handshakes and baby wrappers in public places. Therefore, assisting mothers to step aside and breastfeed in separate rooms was a good practice that needs to be strengthened during training of community volunteers. A broader contextualisation of local understanding of health benefits of some cultural practices related to breastfeeding can be used in future community-based PMTCT programmes. In such approaches, the information on hygienic practices during breastfeeding, risks of wet nursing, risks of mixed feeding of breastmilk with other liquids and solids in the first six months of the infant’s life and others identified in similar settings could be packaged for health education on infant and young child feeding with best known evidence-based alternatives for orphaned babies. This, however, calls for additional resources to support CBVs with materials and allowances when conducting the community educational campaigns and home visits because there is a cost involved even in informal care.^31^

## Study limitations

Lack of empirical evidence on prevalence of exclusive breastfeeding in the settings studied had a bearing on conclusions made on breastfeeding practices. We also recognised the lack of generalisation of the findings beyond the group studied; however, this did not weigh down the value of research findings to inform interventions on integration of community volunteers in PMTCT programmes to promote breastfeeding among HIV-positive mothers in this and similar settings.

## Conclusion

We conclude that the role of CBVs in the implementation of PMTCT interventions at community level can be strengthened by improving the training and development of appropriate educational materials that are sensitive to cultural norms and practices in this setting. Therefore, using a community-driven package of PMTCT intervention is feasible in low-resource settings, where voluntary work is already embraced at the lowest level of service delivery. Future research aimed at determining HIV-free survival of exposed infants in this and similar settings can draw lessons from this study and use community approaches to improve uptake of PMTCT interventions.
